# Accurate variant detection across non-amplified and whole genome amplified DNA using targeted next generation sequencing

**DOI:** 10.1186/1471-2164-13-500

**Published:** 2012-09-20

**Authors:** Abdou ElSharawy, Jason Warner, Jeff Olson, Michael Forster, Markus B Schilhabel, Darren R Link, Stefan Rose-John, Stefan Schreiber, Philip Rosenstiel, James Brayer, Andre Franke

**Affiliations:** 1 Institute of Clinical Molecular Biology, Christian-Albrechts-University, Kiel, Germany; 2 RainDance Technologies, Inc. Lexington, Massachusetts, USA; 3 Institute of Biochemistry, Christian-Albrechts-University, Kiel, Germany; 4 First Medical Clinic, University Hospital, Kiel, Schleswig-Holstein, Germany

**Keywords:** High-throughput targeted next-generation resequencing, Microdroplet-based multiplex PCR, Sample pooling or multiplexing, Whole-genome amplified DNA samples, Cost reduction

## Abstract

**Background:**

Many hypothesis-driven genetic studies require the ability to comprehensively and efficiently target specific regions of the genome to detect sequence variations. Often, sample availability is limited requiring the use of whole genome amplification (WGA). We evaluated a high-throughput microdroplet-based PCR approach in combination with next generation sequencing (NGS) to target 384 discrete exons from 373 genes involved in cancer. In our evaluation, we compared the performance of six non-amplified gDNA samples from two HapMap family trios. Three of these samples were also preamplified by WGA and evaluated. We tested sample pooling or multiplexing strategies at different stages of the tested targeted NGS (T-NGS) workflow.

**Results:**

The results demonstrated comparable sequence performance between non-amplified and preamplified samples and between different indexing strategies [sequence specificity of 66.0% ± 3.4%, uniformity (coverage at 0.2× of the mean) of 85.6% ± 0.6%]. The average genotype concordance maintained across all the samples was 99.5% ± 0.4%, regardless of sample type or pooling strategy. We did not detect any errors in the Mendelian patterns of inheritance of genotypes between the parents and offspring within each trio. We also demonstrated the ability to detect minor allele frequencies within the pooled samples that conform to predicted models.

**Conclusion:**

Our described PCR-based sample multiplex approach and the ability to use WGA material for NGS may enable researchers to perform deep resequencing studies and explore variants at very low frequencies and cost.

## Background

Researchers have undertaken large genome-wide association studies (GWAS), linkage analyses and candidate gene studies to elucidate underlying variations to better understand complex biological paradigms. These individual genotyping approaches typically explain up to 25% [1] of the heritable risk of common, complex polygenic disease and usually miss potentially relevant rare alleles [2]. Nevertheless, GWAS have identified many regions that require additional studies to further identify the causative variants within them. Recently, the advent of next-generation sequencing (NGS) technologies [3] has enabled researchers to catalogue and estimate the contribution of many different types of medically important genetic variants (common and rare SNPs, insertions and deletions, known and novel variants) that have the potential to explain new aspects of the not yet identified heritability [4,5].

NGS technologies clearly have a higher throughput than traditional Sanger sequencing at a significantly lower per base cost, enabling the reading of whole genome(s) in a short period of time [6,7]. Nevertheless, whole genome sequencing is not time or cost effective if the researcher is only interested in a small percentage of the genome in a large number of samples. Therefore, numerous targeted NGS (T-NGS) approaches have been developed to allow selecting and enriching intended regions from a DNA sample before entering into the NGS pipeline [7,8]. By selective recovery and sequencing of only genomic regions of interest, a T-NGS approach can be more cost-effective and result in considerably less weighty data to handle and analyze [8]. Several standardized T-NGS approaches exist that allow whole exome sequencing for Mendelian disorders [9]. Additionally, special-purpose and customized T-NGS approaches allow gene loci of interest to be analyzed that are not sufficiently addressed by exome approaches/kits.

The targeted enrichment methods described so far fall mainly into three categories: hybridization capture, PCR/amplification-based and selective circularization approaches. These methods differ in several aspects such as the use of solution versus solid-phase hybridization, probe design, cost per sample and workflows with implications for automation. In fact, each method has its own advantages and disadvantages [7,8,10,11]. In choosing a target-enrichment approach there can also be many points to consider and balance, such as genomic complexity and nature of the region of interest, study specific needs and objectives, desired fold enrichment, specificity as well as available budget.

When ‘indexing’ strategies are applicable during the process [12-14], a multiplex T-NGS approach can enable the researcher to efficiently leverage the massive throughputs offered by NGS platforms and process more samples in parallel. Molecular barcoding protocols can be used to index different samples together within the same sequencing run. In doing so, the sequencing costs can be amortized across each sample resulting in lower per sample sequencing costs and the ability to quickly process enough samples to achieve the necessary statistical power in an experimental design.

Genomic DNA (gDNA) is often a limiting resource and whole genome amplified (WGA) gDNA is usually prepared/used within many human disease studies to preserve the original precious resources. However, the effect of WGA gDNA on variant discovery from T-NGS data is unclear and needs careful evaluation [8]. To process WGA samples we need to use a target-enrichment method that shows a high specificity in order to tackle, to some extent, bias issues that can be introduced during WGA preparation. The recent benchmark experience with the leading target-enrichment technologies [8,10] concluded that both PCR- and circularization-based methods are superior to hybrid capture methods with respect to achieved specific and uniform sequence coverage, albeit they are not able to target large regions in a single experiment, which is in contrast an advantage of hybrid capture approaches.

To this end, the objective of this work was to (1) evaluate the accuracy of variation discovery within T-NGS data obtained from matched non-amplified and preamplified WGA HapMap gDNA samples, and (2) test the effect of sample multiplexing for cost- and time-saving reasons, at different stages of the sequencing workflow, on key enrichment metrics. We targeted 384 discrete exons involved in cancer [15] using a combined T-NGS workflow of a microdroplet-based PCR sample enrichment pipeline (RainDance Technologies, Inc. USA) and a NGS technology platform (SOLiD, Life Technologies, USA). The results suggest that using WGA as a starting material and pooling PCR-based enriched samples before sequencing don’t substantially impact on the performance of the analyses.

## Methods

**Figure 1:**
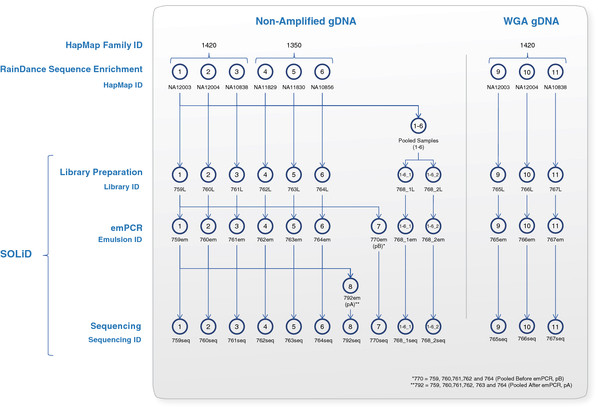
**Experimental Workflow and Study Design.** This Figure shows the tested samples (individual, pooled, non-amplified gDNA, and matched WGA) using the established T-NGS workflow (for more details see Additional file 3: Table S1). Each enriched individual sample was barcoded and sequenced under the following conditions: 1) one sample per octet, 3) samples were pooled after SOLiD library construction, pre-emulsion PCR (emPCR), and 3) samples were pooled post-emPCR. To assess the reproducibility of the established T-NGS method, equimolar amounts of the enriched six HapMap samples were also pooled before SOLiD library construction and tested in duplicate (Libraries ID 768_1L and 768_2L are technical replicates).

The here established workflow and study design are shown in Figure 1.

### Samples tested

In this study we evaluated six HapMap gDNA samples (Coriell Repositories, USA), members of two family trios (IDs 1350 and 1420). We tested samples IDs NA11829, NA11830, and NA10856 of family ID 1350, and NA12003, NA12004, and NA10838 of family ID 1420. Whole-genome amplification of the same three gDNA samples of family ID 1420 was prepared following standard protocol of GenomiPhi V2 Amp Kit 500 RXS (Amersham Biosciences; GE Healthcare Europe GmbH). In each WGA reaction, one μl gDNA (concentration 10 ng/μl) served as a start material. A typical WGA gDNA yield of 4–7 μg gDNA (~ 250 ng/μl) with average product length of >10 kb was achieved. After verifying the quality of the DNA/WGA samples, the enrichment of the intended target regions of all nine samples, six non-amplified gDNA and three matched WGA gDNA, were undertaken using droplet-based multiplex PCR (RainDance; USA) as described below.

### Targeted genomic sequences and primer panel design

The RainDance Technologies “384 Member Primer Panel” targets 384 Consensus CDS exons from 373 different genes thought to contain somatic mutations involved with cancer [15]. Amplicons were designed as described by Sjoblom et. al. The amplicons contained in the RainDance “384 Member Primer Panel” represent 172,053 amplicon bases (Additional file 1: Table S2). Amplicons were selected to represent several different design parameters that include: amplicon length (300 to 600 bases), amplicon GC content (30 – 60%) and Primer T_m_ (56 - 60°C). The RainDance “384 Member Primer Panel” contains 384 unique primer droplets, one for each amplicon in the panel. The forward and reverse primers for each amplicon are synthesized and combined into a single primer aliquot in the well of a standard microtiter plate. Each primer droplet contains an equal concentration of the forward and reverse primer (5.2 μM per primer). The primer aliquot is then reformatted to create an emulsion containing many primer droplets that are identical to each other, each containing a single primer pair. The primer droplets are 25 μm in diameter with a volume of eight pL per droplet. Each primer droplet is evaluated for its size and morphology to ensure each primer is represented at the same concentration. An automated counting process quantifies the number of primer droplets generated from each primer aliquot. The different primer droplets are then mixed together to ensure that each primer panel has the same number of each unique primer droplet.

### Genomic DNA fragmentation

Genomic DNA samples were fragmented using a nebulization kit (Invitrogen, K7025-05) following the manufacturer’s recommended protocol: 2.0 μg of gDNA was re-suspended in 750 μL Shearing Buffer (TE, pH 8.0 (Fisher, 50843207) containing 10% glycerol (Fisher, AC15892)) and was nebulized at 6–10 lb per square inch (psi) for 90 s to produce 2–4 kb DNA fragments. Fragmentation of the gDNA to 2–4 kb produces the optimal template size for the amplicons size distribution represented in the RainDance “384 Member Primer Library”. Sheared gDNA was precipitated by adding 80 μL 3 M sodium acetate, pH 5.2 (Fisher, 50843081), 4 μL 20 mg/ml Mussel Glycogen (Fisher, NC9329100) and 700 μL 100% isopropanol (Fisher, AC14932) mixed and stored overnight at −20°C. The samples were then centrifuged at the maximum speed for 15 min at 4°C. The supernatant was removed, 500 μL of cold 80% ethanol (Fisher, 5739852) wash buffer was added and the DNA pellet was spun down by centrifugation at the maximum speed for 5 min at 4°C. The pellet was air dried and re-suspended in 10 μL 10 mM Tris–HCl, pH 8.0 (Sigma, T2694). Fragmented genomic DNA was run on a 0.8% agarose gel to confirm that the genomic DNA was in the correct size range (2–4 kb), data not shown.

### Genomic DNA template Mix

In order to prepare the gDNA Template Mix, 1.5 μg of the purified fragmented gDNA was added to 4.7 μL 10× High-Fidelity Buffer (Invitrogen, 11304–029), 1.26 μL of MgSO4 (Invitrogen, 11304–029), 1.6 μL 10 mM dNTP (New England Biolabs, NO447S/L), 3.6 μL Betaine (Sigma, B2629-50 G), 3.6 μL of RDT Droplet Stabilizer (RainDance Technologies, 30–00826), 1.8 μL dimethyl sulfoxide (Sigma, D8418-50 ml) and 0.7 μL 5 units/μL of Platinum High-Fidelity Taq (Invitrogen, 11304–029) the samples was brought to a final volume of 25 μL with Nuclease Free Water, Teknova (Fisher, 50843418).

### Merge genomic DNA template Mix and primer panel droplets: RDT 1000 instrument

PCR droplets were generated on the RDT 1000 (RainDance Technologies, Inc. USA) one droplet at a time using the manufacturer’s recommended protocol. All of the resulting PCR droplets were automatically dispensed as an emulsion into a single PCR tube and transferred to a standard thermal cycler for PCR amplification. Each sample generated an emulsion containing more than 1,000,000 droplets in which each droplet contained a single-plex polymerase chain reaction (PCR) that targeted one of the 384 targets defined by the RainDance Technologies “384 Member Primer Panel”. Each amplicon was represented by multiple unique PCR droplets (Additional file 2: Figure S1).

### PCR amplification

Samples were cycled in a Bio-Rad PTC-225 thermal cycler with the following profile: 94°C for 2 min; 55 cycles at 94°C for 15 s, 58°C for 15 s, 68°C for 30 s; 68°C for 10 min and hold at 4°C.

### Breaking emulsion

After PCR Amplification the emulsion of PCR droplets were broken, to release each individual amplicon from the PCR droplets, using the manufacturer’s recommended protocol.

### PCR product clean Up

Each sample was purified over a MinElute column (Qiagen, 28004) following the manufactures recommended protocol. The sample was eluted off the column with 11 μL of the Qiagen Elution Buffer. The purified amplicon DNA was then run on an Agilent Bioanalyzer to confirm that the amplicon profile (Additional file 2: Figure S2 A) matches the predicted histogram distribution (Additional file 2: Figure S2 B).

### SOLiD sequencing library construction and sample indexing

The sequencing libraries were constructed according to the Standard Fragment Library preparation of the SOLiD 3.0 system protocols (Applied Biosystems, USA). Six individual sequencing libraries were prepared using the enriched non-amplified gDNA PCR products and indexed with barcodes 1 to 6 (Libraries IDs 759L-BC1, 760L-BC2, 761L-BC3, 762L-BC4, 763L-BC5 and 764L-BC6, respectively). Three other libraries were prepared using the enriched WGA gDNA products and indexed with barcodes 9, 10 and 11 (Library IDs 765L-BC9, 766L-BC10 and 767L-BC11, respectively) (Figure 1 and Additional file 3: Table S1). In parallel, to test the performance of sample pooling before library preparation, equimolar portions of each of the six non-amplified gDNA enriched products (based on final concentration of each sample) were pooled together in duplicates and library IDs 768_1L and 768_2L were prepared. In total, 11 Standard Fragment Libraries were prepared (six individual gDNAs, three individual WGAs, and two pools, which comprised two duplicate pools of the six non-amplified gDNA enriched products) (details in Additional file 3: Table S1). For each library 0.5-1.0 μg enriched gDNA/WGA/pool was used.

### Purification of the SOLiD libraries

After library preparation, sizing, quantification and quality control of the prepared SOLiD libraries were performed on Agilent Bioanalyzer 2100 (Agilent Technologies, Waldbronn, Germany). A typical electropherogram obtained (Additional file 2: Figure S3 A) showed a primer-dimer peak between 60 and 70 bp (~68 bp). These primer-dimers, and other unincorporated dNTPs, primers, salts and other contaminants, were removed and PCR amplicons >100 bp were recovered according to the standard procedure of Agencourt AMPure Kit (Beckman Coulter Genomics). Removal of the primer-dimers from the SOLiD library construction and sample indexing step was necessary to reduce the carryover of these products into the library. After the purification step, sizing and quantification of all the generated libraries were measured again on the Agilent 2100 Bioanalyzer (Additional file 2: Figure S3 B) shows a typical electropherogram after purification).

### Pooling strategies of indexed samples

We evaluated here the performance of sample indexing and pooling before and after the emPCR step. After library purification, emPCR was carried out for three different scenarios (summarized in Figure 1): A) Nine individual emPCRs were carried out for the non-amplified gDNA’s (six reactions; emulsion IDs 759em, 760em, 761em, 762em, 763em and 764em) and the WGA gDNA’s (three reactions; emulsion IDs 765em, 766em and 767em). B) Two emPCR reactions were carried out from the two pooled samples before library construction: emulsion IDs 768_1em and 768_2em. C) Equimolar portions of five of the non-amplified gDNA libraries (IDs 759L, 760L, 761L, 762L and 764L) were collected and one emPCR was performed (emulsion ID 770em (pB)). Library ID 763L with BC 5 was not pooled with the afore-mentioned five gDNA libraries due to its low concentration, which was only appropriate for one emPCR of the individual library ID 763em. Finally, after performing all the afore-mentioned emPCR reactions, a single pool of equimolar amounts of the obtained individual emPCR products from non-amplified gDNA emulsion IDs 759em, 760em, 761em, 762em, 763em and764em was generated and processed in parallel (emulsion ID 792em (pA)). Therefore, a total of 13 emPCR reactions were performed for all the tested comparisons (details in Additional file 3: Table S1 and Additional file 2: Figure S1).

### NGS using SOLiD 3.0 system platform

The sequencing was carried out on the SOLiD 3.0 system platform (Applied Biosystems/Life Technologies, MA, USA) using Standard Fragment Library protocol and lengths of 50 bp according to the manufacturer’s instructions (Applied Biosystems, MA, USA). The principle steps of the SOLiD sequencing protocol are well covered [11,16-18]. Here each enriched product (single or pooled libraries) was run on a single well/spot of an octant slide. A total of 13 wells/spots were used (759seq, 760seq, 761seq, 762seq, 763seq, 764seq, 765seq, 766seq, 767seq, 768_1seq, 768_2seq, 770seq and 792seq), i.e. one complete octant slide (8/8) and five spots (two-thirds) of a second octant slide (5/8), for these experiments to sequence the 13 different sequencing samples (listed in Additional file 3: Table S1 and Additional file 2: Figure S1). The SOLiD platform interrogates each base by two separate probe hybridizations (i.e. two color calls), which has the advantage of improved base calling accuracy (invalid color call sequences point to sequencing errors). The color space reads are translated into (base space) fasta code during alignment to a reference genome, which is performed off the machine, on a high performance computer cluster.

### Mapping the SOLiD sequencing data

SOLiD sequencing reads (50 bp) were aligned to the human genome (hg18/NCBI36) using the CLCbio Genomics Workbench (v 3.0) [19] with a custom RainDance Workflow plug-in, and in-house Perl scripts. Before assembly, the reference genome was annotated with the 384 amplicon targets, consisting of 382 discontiguous regions, using a gff file (Additional file 1: Table S2). Primer locations of non-overlapping amplicon targets were omitted from analysis as SNP detection is not possible under the primer as the PCR will generate amplicon product for these regions from the primer and not the gDNA. Default settings for reference assembly (Additional file 3: Table S3) were used. Non-unique reads were mapped randomly to possible placements. All aligned sequence reads were exported in SAM [20] format.

### Sequence variant identification

Single nucleotide sequence variants were identified using the CLC bio Genomics Workbench (v 3.0), using default settings with the following exceptions: minimum allele frequency was set to 10%, minimum coverage was set to 5, maximum coverage was set to 15,000. Only SNPs that were located in amplicon target regions, excluding primer locations, were analyzed. Nucleotide coverage of known SNP positions in the target regions were extracted using a Perl wrapper in combination with Samtools 0.1.7 [20]. Samtools pileup files were combined with a Perl script. Nucleotide sequence data reported are available in the GenBank databases (the Sequence Read Archive (SRA)) under the study accession number ERP000999 [21]).

### Data analysis

SNP concordance was determined by comparing HapMap genotype data (HapMap Public Release #27, merged II + III) to SNPs found using the CLC bio Genomics Workbench. Caucasian (CEU) HapMap SNPs from NA12003, NA12004, NA10838, NA11829, NA11830, and NA10856 were downloaded from [22]. SNPs detected from the SOLiD sequencing data were also compared to SNPs from dbSNP, Build 130. A Perl script was written to extract all known SNPs from the six HapMap samples.

SNPs were considered heterozygous if non-reference allele frequency was 10-90%. SNPs were considered homozygous if non-reference allele frequency was equal to or greater than 90%. For a SNP to be considered detected, the SNP had to have at least five reads per allele. Therefore a heterozygous SNP had to have at least 10 counts per position to be considered “detected”. Only detected SNPs were used for concordance calculations.

## Results

The experimental workflow and investigated samples are summarized in Figure 1. Several experimental alternatives were performed to investigate the effect of using WGA gDNA material and different pooling strategies on the performance of selective recover of genomic sub-regions of interest and on variant detection.

### Sample throughput, sequence capacity and enrichment metrics

Genomic alignment and SNP detection were carried out as described in Methods. On average, 66% of the sequencing reads that aligned to the human genome (that were 30-40% of total raw reads), mapped to the target regions. This on-target percentage, or specificity, appeared unaffected by the pooling strategy used. However, a slight decrease in specificity was observed in the WGA samples. For example, specificity of sample NA12003, NA12004, and NA10838 dropped from 65.3%, 73.3%, and 66.0% for non-amplified gDNA to 63.2%, 64.4%, and 57.8%, respectively, for the WGA samples.

The C1 coverage was high (>99%) across all of the non-pooled samples indicating a success of each primer pair to produce an amplicon. All of the expected amplicons in this panel (383/383) produced a PCR product. Amplicons with low coverage might result from the inability for the sequencing chemistry to sequence through the sequence context of the amplicon.

Although coverage at 20**×** (C20) varied, it correlated to mean base coverage as expected. However, normalized base coverage (C0.2× of the mean) was consistent throughout all the samples (85.6% ± 0.6%), suggesting that the utilized enrichment process is robust and reproducible. For samples with mean base coverage of at least 200 reads per base, C20 was at least 90%. For all samples that had a mean target base coverage of 200 reads per base, the percentage of bases that were covered at least once (C1) was greater than 98%. Also, C1 increased to 99.4-99.7% when mean base coverage was greater than 1000×. In addition, the results not only showed adequate average coverage and enrichment folds for reliable SNP discovery, but also adequate uniform coverage of the barcoded and pooled samples before and after emPCR (Additional file 2: Figure S4). A low number of reads of the samples indexed with BC4 was observed (gDNA-pB 770- BC4 and gDNA-pB 792- BC4; Table 1), compared to all other indexed samples with other barcodes. Consequent lower coverage (Table 1) and SNP detection rates (Table 2) were also observed – while the concordance rates were not similarly affected. The substantial underrepresentation of samples indexed with BC4 has also been revealed in other in-house multiplex experiment including BC4 on human gDNA samples using different sample enrichment technologies (see Discussion).

**Table 1:** Sample throughput and sequence capacity- general sequencing and enrichment metrics

**HapMap sample ID**	**Sample type**	**Library ID**	**Reads**	**Mapped**	**On-target %**	**ADoC**	**C1**	**C20**	**Coverage 0.2X mean**
**NA12003**	**gDNA**	**759L**	36,453,208	33.50%	65.30%	2,121	99.50%	98.00%	86.10%
	**WGA**	**765L**	42,752,716	36.30%	63.20%	2,588	99.60%	98.10%	86.40%
	**gDNA-pB**	**770L_BC1**	10,031,809	36.40%	64.10%	621	99.00%	96.50%	86.50%
	**gDNA-pA**	**792L_BC1**	5,221,822	31.60%	64.20%	280	98.40%	93.50%	85.70%
**NA12004**	**gDNA**	**760L**	39,005,646	31.00%	73.30%	2,356	99.40%	98.00%	86.30%
	**WGA**	**766L**	43,079,257	17.50%	64.40%	1,273	99.40%	97.20%	84.80%
	**gDNA-pB**	**770L_BC2**	3,272,424	39.30%	72.70%	249	98.30%	93.60%	87.10%
	**gDNA-pA**	**792L_BC2**	6,023,113	32.50%	72.90%	380	98.50%	94.60%	85.70%
**NA10838**	**gDNA**	**761L**	35,573,703	21.10%	66.00%	1,309	99.40%	97.50%	85.90%
	**WGA**	**767L**	43,140,968	30.60%	57.80%	2,014	99.60%	98.20%	86.50%
	**gDNA-pB**	**770L_BC3**	7,114,637	35.80%	67.50%	456	98.60%	95.30%	85.80%
	**gDNA-pA**	**792L_BC3**	6,587,601	34.00%	67.60%	401	98.60%	94.90%	85.80%
**NA11829**	**gDNA**	**762L**	39,864,620	29.20%	63.90%	1,961	99.60%	97.90%	85.50%
	**gDNA-pB**	770L_BC4*	638,985	36.40%	66.00%	41	95.40%	63.40%	84.60%
	**gDNA-pA**	792L_BC4*	677,767	28.50%	66.40%	34	94.50%	55.40%	84.90%
**NA11830**	**gDNA**	**763L**	38,654,830	34.10%	68.50%	2,404	99.70%	98.10%	85.80%
	**gDNA-pB**	**770L_BC5**	Insufficient amount of material to run sample						
	**gDNA-pA**	**792L_BC5**	5,403,560	32.80%	68.10%	320	98.40%	94.10%	85.60%
**NA10856**	**gDNA**	**764L**	43,185,707	34.60%	61.10%	2,418	99.50%	98.00%	85.80%
	**gDNA-pB**	**770L_BC6**	9,255,333	36.30%	61.30%	545	98.90%	96.00%	85.90%
	**gDNA-pA**	**792L_BC6**	6,435,690	33.90%	62.00%	357	98.50%	94.40%	85.00%
**Pooled Samples**	**gDNA**	**768L_1**	41,913,860	32.40%	67.00%	2,406	99.80%	98.30%	84.90%
	**gDNA**	**768L_2**	41,958,628	33.40%	66.70%	2,474	99.60%	98.30%	85.30%

- **Reads:** Total sequencing reads per sample.

- **Mapped:** Percentage of total reads that could be aligned to the human genome (hg18/NCBI).

- **On-Target:** Percentage of mapped reads that align to the target regions.

- **ADoC:** Average depth of coverage of target base.

**- C1:** Percentage of target bases that are covered by at least one sequencing read.

- **C20:** Percentage of target bases that are covered by at least 20 sequencing reads.

- **Coverage 0.2× Mean:** Percentage of target bases that are covered by at least 0.2× of ADoC. Note that one barcode (BC4) was underrepresented (assigned with “*” in this Table; see also Results and Discussion sections).

- **gDNA:** genomic DNA; **WGA:** whole-genome amplification; **emPCR:** emulsion PCR; **BC:** barcode.

**Table 2:** Variant detection and concordance- SNP concordance with HapMap genotypes

**HapMap sample ID**	**DNA type**	**Library ID**	**Detection**	**Concordance**	**Homozygous**	**Heterozygous**	**SNPs with genotypes**	**SNPs not detected**	**False negative detection rate**
**NA12003**	**gDNA**	**759L**	98.20%	100.00%	100.00%	100.00%	268	0	0,0%
	**WGA**	**765L**	97.80%	100.00%	100.00%	100.00%	268	0	0,0%
	**gDNA-pB**	**770L_BC1**	97.40%	100.00%	100.00%	100.00%	268	2	0,7%
	**gDNA-pA**	**792L_BC1**	96.50%	100.00%	100.00%	100.00%	268	5	1,9%
**NA12004**	**gDNA**	**760L**	99.10%	99.10%	99.10%	100.00%	138	0	0,0%
	**WGA**	**766L**	99.10%	99.10%	99.10%	100.00%	138	1	0,7%
	**gDNA-pB**	**770L_BC2**	98.30%	99.10%	99.10%	100.00%	138	3	2,2%
	**gDNA-pA**	**792L_BC2**	97.40%	99.10%	99.10%	100.00%	138	3	2,2%
**NA10838**	**gDNA**	**761L**	99.10%	99.10%	98.90%	100.00%	270	2	0,7%
	**gDNA-pB**	**770L_BC3**	98.70%	99.60%	99.50%	100.00%	270	3	1,1%
	**gDNA-pA**	**792L_BC3**	98.30%	99.60%	99.50%	100.00%	270	2	0,7%
**NA11829**	**gDNA**	**762L**	97.90%	100.00%	100.00%	100.00%	274	2	0,7%
	**gDNA-pB**	**770L_BC4**	91.0%*	99.50%	99.50%	100.00%	274	43	15,7%
	**gDNA-pA**	**792L_BC4**	86.3%*	100.00%	100.00%	100.00%	274	55	20,1%
**NA11830**	**gDNA**	**763L**	99.10%	99.10%	100.00%	93.90%	272	0	0,0%
	**gDNA-pB**	**770L_BC5**	Insufficient amount of material to run sample						
	**gDNA-pA**	**792L_BC5**	98.70%	99.10%	100.00%	94.10%	272	9	3,3%
**NA10856**	**gDNA**	**764L**	99.60%	99.60%	100.00%	97.40%	273	1	0,4%
	**gDNA-pB**	**770L_BC6**	98.70%	99.10%	99.50%	97.30%	273	3	1,1%
	**gDNA-pA**	**792L_BC6**	99.10%	99.60%	100.00%	97.40%	273	4	1,5%

- **Detection:** Percentage of SNPs that are covered by at least five sequencing reads.

- **Concordance:** Percentage of detected SNPs that matched the HapMap (HapMap Public Release #27; merged II + III) genotype. Due to the underrepresentation of barcode 4 (BC4) in the pools 770 and 792, the detection and concordance rates were lower (assigned in this Table with *).

- **gDNA:** genomic DNA; **WGA:** whole-genome amplification; **emPCR:** emulsion PCR; **BC:** barcode.

Altogether, the results shown in Table 1 demonstrate good performance on both non-amplified gDNA and WGA gDNA samples with no substantial difference in the sequence performance among the samples and reveal specificity of 66.0% ± 3.4% and uniformity (Coverage at 0.2× of the mean) of 85.6% ± 0.6%.

As a higher coverage depth is a pre-requisite for somatic and rare variant identification in particular, the samples were re-analyzed to calculate the coverage beyond 20-fold (C20) in our experimental design (Additional file 3: Table S4). The C30, C50 and C100 for the individual samples, which were each sequenced in a separate octet (759L, 760L, 761L, 762L, 763L and 764L), maintained high levels of average coverage across the target region (C30 = 97.76%, C50 = 97.24% and C100 = 96.08%). The individually barcoded samples expectedly showed lower coverage at C30, C50 and C100 (average coverage C30 = 94.31%, C50 = 91.54% and C100 = 83.87%). These results suggest that this T-NGS methodology may be applied for the analysis of genetic variations in cancer genes.

### Variant detection and concordance - SNP concordance with HapMap genotypes

To determine the concordance of the variant detection, the generated sequences per HapMap sample were compared to their reference genotypes and across the different sample treatments (non-amplified gDNA, WGA gDNA, single and pooled). Summary concordance data is shown in Table 2. In total 333 SNPs were compared and false negative detection rates were estimated (Table 2). In general, the results shown in Table 2 indicate low false negatives (0.0 to 3.3%). Individually sequenced samples showed a modestly lower false negative detection rate (0.3% and 0.2%, for non-amplified gDNA and WGA, respectively) than that of the corresponding pooled gDNA and WGA gNDA paired samples (1.3 to 1.9%). The SNP detection rate correlated with the mean base coverage, and was unaffected by the pooling strategy. Importantly, the WGA gDNA samples showed nearly similar concordance rate to that of the non-amplified gDNA samples (Table 2). Overall, concordance averaged 99.5% and generally remained unchanged, regardless of the applied pooling strategy.

### Efficiency and detection SNP rates of Non-barcoded and pooled samples

The sequencing results of the two technical replicates (samples 768_1L and 768_2L) were used to analyze different enrichment measures, variant detection rates, and the reproducibility of the method. Samples 768_1L and 768_2L were pooled from sample ID’s 1, 2, 3, 4, 5 and 6 and represented HapMap samples NA12003, NA12004, NA10838, NA11829, NA11830, and NA10856, respectively (Figure 1). Since genotyping data from NA12004 was limited for all positions that were considered, genotypes were inferred from the non-barcoded samples and used for comparison. Because we were inferring genotypes from the sequencing data, it allowed us to generate a larger SNP set which also included non-HapMap and non-dbSNP (build 130) SNPs. These non-dbSNP SNPs could represent valid, potential novel SNPs.

This strategy to infer SNPs with confidence stemmed from the knowledge that we have selected samples from HapMap trios and the inferred SNPs followed Mendelian patterns of inheritance within each trio. Furthermore the inferred SNPs were observed and concordant between replicate samples built into our experimental design (individual samples, pooled samples and WGA samples) for HapMap family 1420.

After genotypes were calculated for these positive control SNPs (i.e. SNPs validated across multiple samples), a cumulative genotype was calculated for the pool. SNP detection in the non-barcoded, pooled samples were such that the non-reference allele had to represent at least 1% of the total allele count for a given nucleotide. The minimum number of counts for each non-reference allele was varied (5, 10, and 20) to optimize sensitivity and specificity (Table 3). The lowest false SNP discovery rate (~10%) in the technical replicates was detected at a stringent coverage parameter (20×). The reverse holds true at lower (5×) coverage; we reached 40% false discovery rate (Table 3).

**Table 3:** Efficiency and SNP detection rates of non-barcoded and pooled samples

**Minimum read count for SNP call**	**Library ID**	**Positive control SNPs**	**Positive control SNPs in sample**	**Total SNPs in sample**	**Sensitivity**	**False discovery rate**
**5**	**768_1L**	244	226	376	92,6%	39,9%
	**768_2L**	244	230	371	94,3%	38,0%
**10**	**768_1L**	244	212	277	86,9%	23,5%
	**768_2L**	244	212	267	86,9%	20,6%
**20**	**768_1L**	244	193	214	79,1%	9,8%
	**768_2L**	244	198	221	81,1%	10,4%

- **Minimum read count for SNP call**: Minimum number of non-reference allele counts required for a SNP to be considered detected.

- **Positive control SNPs**: Positive control SNPs generated from the non-pooled, non-barcoded data (759–764). Since the HapMap genotyping data was incomplete, even for known SNPs, we attempted to create a positive control set of SNPs within the targeted regions. If the SNP was detected within samples 759–764, a combined genotype was determined for that SNP position. For example, position X was determined to have a “CG” genotype in sample 759 and position X had the reference genotype of “CC” in samples 760–764, the predicted allele frequency would be 8.3% (1 in 12). In the non-pooled samples, a SNP with a non-reference allele frequency of 10-90% was considered a heterozygote. A homozygous SNP in non-pooled samples was defined as having >90% non-reference allele frequency. The number in this column represents the total number of SNPs that have a non-reference allele within a given pooled sample. Note that these positive control SNPs include HapMap samples with rs IDs, non-HapMap samples with rs IDs, and potentially novel SNPs.

- **Positive control SNPs found**: This number represents the number of positive control SNPs that were detected in a given pool with a given set of parameters.

- **Total SNPs detected**: This number represents the total number of SNPs found in a given pool with a given set of parameters. This number contains the “positive SNPs found” plus other SNPs. It is assumed that most of these SNPs are false positives since this number decreases significantly if you increase the stringency of your SNP detection parameters. However, some novel SNPs could exist in this set.

- **Sensitivity**: In this case, this is simply the percentage of positive controls SNPs found in a given pool with a given set of parameters. Sensitivity decreases as SNP detection stringency increases.

- **False Discovery Rate**: This was defined as (total SNPs detected – positive control SNPs found)/Total SNPs detected * 100.

**Figure 2:**
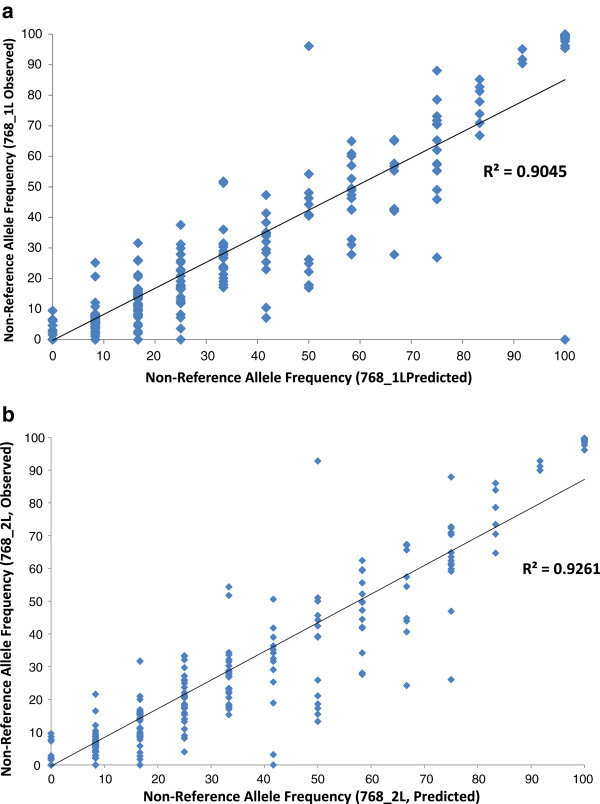
**(a and b) Actual Versus Predicted Allele Frequencies in Pooled Samples.** These two Figures show the correlation between predicted (X-axis) and observed (Y-axis) non-reference allele frequencies in the two pooled gDNA samples before SOLiD library preparation, namely sample/library 768_1L (replicate 1; Figure 2a) and 768_2L (replicate 2; Figure 2b). For each positive control SNP, a genotype was inferred from the non-pooled sequencing samples. Composite SNP allele frequencies were calculated for each pool and compared to the actual SNP allele frequencies. R^2^ values represent the square of the Pearson coefficient and reveal good correlation between samples (0.9045 and 0.9261 of sample 768_1L and 768_2L, respectively).

**Figure 3:**
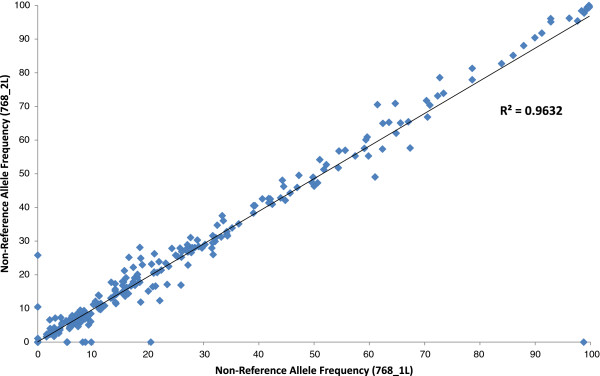
**Reproducibility of target enrichment: Library 768_1L vs. Library 768_2L.** Figure 3 shows the comparison of non-reference allele frequency within the two technical replicates (768_1L and 768_2L). R^2^ represents the square of the Pearson coefficient and reveals high consistency (R^2^ = 0.9632), and hence reproducibility, between the two replicates.

Actual allele frequencies in the two pooled samples before library preparation (768_1L and 768_2L) correlated well (Pearson R^2^ = 0.90 and 0.93, respectively) with predicted allele frequencies of the pooled gDNA samples (Figure 2a and 2b). The non-reference allele frequencies were very consistent between the two technical replicates, as illustrated in Figure 3 with high Pearson correlation coefficient (R^2^ = 0.96).

### Minor allele detection and Mendelian inheritance quality check

**Figure 4:**
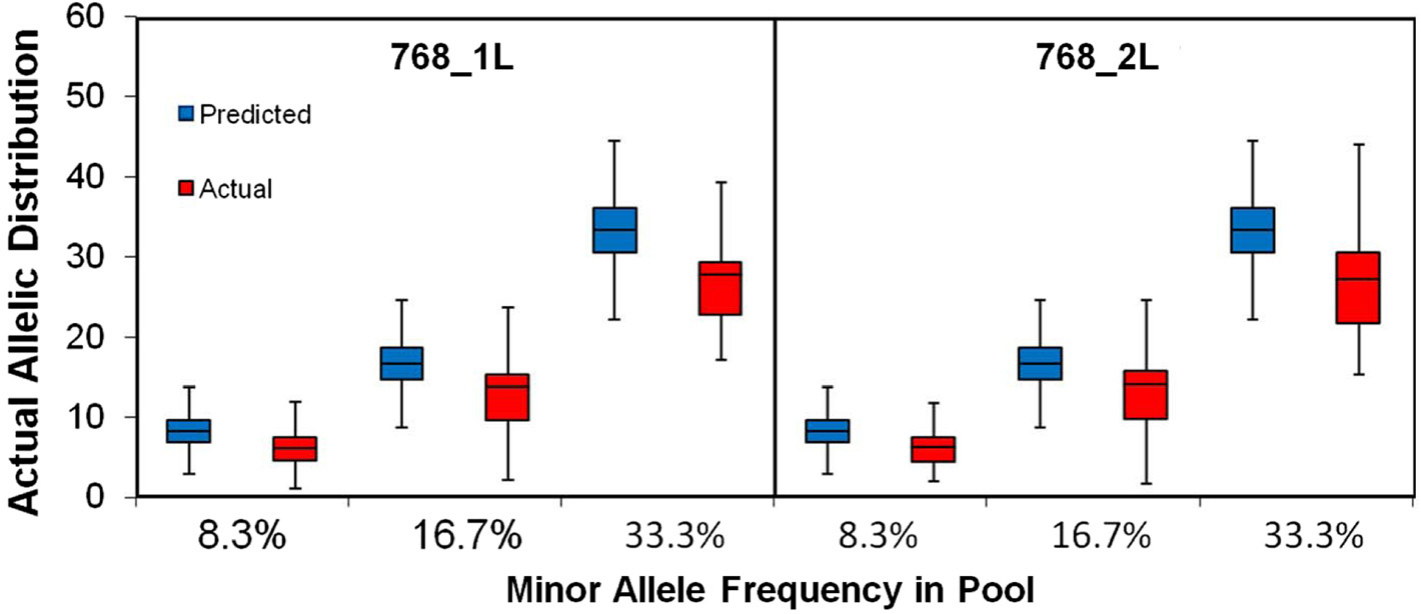
**Pooling of non-barcoded samples to detect rare variants.** Figure 4 shows the ability to detect minor alleles represented in the two technical replicates (pooled samples before library preparation; samples IDs 768_1L and 768_2L), based on the known reference genotypes for each of the individual samples within each pool. In each case, the predicted frequency (blue boxplots) and the actual frequency (red boxplots) were in agreement.

The two pooled samples before library preparation (gDNA samples: Library IDs 768_1L and 768_2L) were also evaluated to detect minor alleles represented in the pool based on the known reference genotypes for each of the individual samples within each pool. The predicted minor allele frequency (MAF) for each sample was calculated by combining the genotypes of each of the six individual samples within the pooled sample. The actual minor allele frequency was calculated for each SNP within the sequenced samples. Boxplots were generated for the three smallest expected MAFs (Figure 4). In each case, the predicted frequency and the actual frequency were in agreement as shown in Figure 4.

In addition, by evaluating family structure, we did not detect any errors in the Mendelian patterns of inheritance of genotypes between the parents and the offspring within each trio (an overview of all SNPs and genotypes detected is shown in Additional file 4: Table S5).

## Discussion

Optimizing T-NGS methods with unbiased performance using non-amplified and amplified (WGA) gDNA samples is necessary to enable statistically powered large-scale studies. Even if the cost of routine sequencing of the human exome and whole-genome becomes affordable in the near future, multiplexed T-NGS will serve alongside as a downstream validation and diagnostic tool to reach sufficient coverage with less sequencing, enabling the investigation of a higher number of samples in parallel. More specifically, T-NGS provides more power to catalogue most types of variations present in clinically relevant subsets of the genome at a fraction of the cost of whole-genome sequencing. Therefore, using mixed approaches, for instance whole-genome sequencing at low-coverage followed by T-NGS at higher coverage, may become more applicable in the coming years [23].

In the present study, we tested the performance of combining a microdroplet-based PCR targeted sequencing approach to NGS and demonstrated its utility in T-NGS of 384 cancer exons throughout the genome of different comparisons. We evaluated the performance of standard gDNA (without amplification) versus whole-genome amplified (WGA) gDNA samples as well as testing different sample indexing strategies from the sequencing workflow, namely pooled samples before library preparation (without sample indexing) versus individual samples, and pre- versus post-emPCR with sample indexing using SOLiD molecular barcodes. The results of T-NGS of 384 cancer exons demonstrate sequencing specificity of 66.0% ± 3.4%, uniformity (coverage at 0.2× of the mean) of 85.6% ± 0.6%, concordance of 99.5% ± 0.4% and no Mendelian inheritance errors. We have also demonstrated the ability to detect minor allele frequencies within pools of six non-barcoded non-amplified gDNA samples. These results show the possibility to process WGA gDNA samples at nearly similar performance to that of standard non-amplified gDNA samples without showing significant allelic bias/difference in enrichment metrics and variation detection among non-pooled and pooled samples. For instance, 95% of the targeted regions in the six HapMap samples that were tested (two different HapMap trios) were covered with at least 20× coverage while maintaining a 99.5% average genotype concordance across all of the samples.

The results show that combining the applied target enrichment approach with NGS technology provided many advantages. This T-NGS approach leverages the sensitivity and specificity of the PCR to efficiently capture and represent the sequence context from different genomic regions [10]. The stringency and flexibility to allocate primers to the targets of interest are required to tackle complex genomic regions of high homology (pseudogenes) and repetitive elements [24-26]. The uniformity of the sequence coverage across all the targeted regions in the indexed samples allows for efficient use of NGS sequencing capacity. Typical uniformity with a panel achieves greater than 85% of bases within 0.2× of the mean coverage. This level of performance allows for predictable sequence coverage beyond what is reported in Table 1 with the appropriate amount of sequencing per sample (see additional coverage analysis in Additional file 3: Table S4). The utilized NGS platform, with its high throughput, allows accurate SNP detection as argued [27] and shown here. Inadequate enrichment and/or coverage depth, which is not the case in our study, can cause failure to detect real nucleotide variation (Table 1; Additional file 4: Table S5), which may lead to higher false-negative rates in particular for heterozygotes [28,29]. In fact, comparable coverage depth and uniformity of the tested 384 exons have been achieved using other NGS technology platforms, 454 FLX and Illumina (Additional file 3: Table S6). As illustrated in Table 2, parallel detection of both homozygous and heterozygous genotypes, points to the efficiency of the tested workflow and indicates that little, if any allelic bias has been introduced during sample enrichment and sequencing processes.

One exception to the observed even distribution of all tested bar-coded samples was the lower representation of samples indexed with BC4. We supposed at first that this might be due to inaccurate quantification and pooling; i.e. lower representation of BC4 in the original libraries before pooling/enrichment, either due to a pipetting error or a wrong DNA concentration measurement during library preparation. Surprisingly, we have also observed the substantial underrepresentation of indexed samples with BC4 in other multiplex experiments using a different sample enrichment technique, hybridization-based sequence capture, in three successive runs [30]. So we considered BC4 as an outlier and we recommend avoiding it in future experiments. In addition, the relatively high SNP false discovery rate (10-40%; Table 3) of the non-barcoded technical replicates (samples 768_1L and 768_2L), although it seems to be dependent to some extent on coverage depth, it indicates that pooling enriched gDNA samples before library construction, using the current T-NGS approach, may not be a sensible option. We have mainly tested this pooling option to access the reproducibility of the established workflow (Figure 3). These results in general emphasize the need to achieve higher coverage to reduce the SNP false discovery rate. Applying of a similar pooling option to enriched WGA samples will likely result in worse data/performance; due to a potential cumulative impact/bias from different DNA amplification reactions. Instead, we recommend pooling the samples before or after emPCR in the course of the NGS protocol (see Table 2 for more details).

Another clear limitation in our study, which needs to be acknowledged, is the relatively low percentage of mapped reads to the human genome (Table 1). The percentage of mapped reads depends on several factors, such as NGS technology platform, target-enrichment approach, mapping approach, and software and analytical approaches. Using the same target region and RainDance amplicon library on different NGS technology platforms resulted in opposite numbers of produced reads and mapping percentages to the human genome (Table 1 and Additional file 3: Table S6). While the long-read NGS platform (454 FLX) generated a lower total number of reads (219,876), the percentage of mapped reads was as high as 84%. In contrast, only up to 40% of the 5 to 44 million reads produced by the short-read NGS platforms (SOLiD and Illumina) mapped to the human genome. This might indicate that shearing of the enriched PCR amplicons during preparation of the sequencing libraries for the short-read NGS platforms (Additional file 2: Figure S3 A and B) leads to generation of fragments that are ambiguously mapped to several locations of the genome (i.e. off-targets). In fact, CLC bio and other mapping tools, such as BWA, randomly map these ambiguous reads to one of the ‘possible’ locations. In addition, we found recently that the quality of SNP calling was substantially affected by the choice of mapping strategy and analytical tools [30]. For example, we showed that the tested widely-used SNP-callers do not seem to be well-trained to handle enrichment data, and thus produced a significant fraction of false positive as well as negative SNP calls. Moreover, changing the mapping settings or using another software version can result in different enrichment metrics. This observation was confirmed here using a newer version of the CLC bio Workbench software (version 5.1), which, for instance, improved the mapping specificity to up to 56% (for details see new Additional file 3: Table S4). The drop in mapping percentage, while apparently worrisome, can still be satisfactory as long as it does not impact on the accuracy of the downstream SNP calling. In other words, achieving a high and even coverage at intended bases (vertical coverage; ≥20×) and good on-target percentage (completeness or horizontal coverage; 60 - 80%), as shown in our study, ensure accurate SNP calling.

The “barcoding” approach holds promise to enable the sequencing of large numbers of samples, and allows for the identification of rare and novel variations in the intended loci as well as variant carrier post-sequencing. Comparing the results of multiplexed barcoded samples pre- and post-emPCR revealed similar performances of both schemes (Table 1, Table 2 and Additional file 2: Figure S6). Therefore we recommend pooling samples before emPCR to save money, time, and effort. Indeed, the best design is to index samples and pool them before enrichment. Testing pre-enrichment sample multiplex was unfortunately infeasible using the applied target-enrichment method and due to the limited length of the generated sequence reads of the utilized SOLiD platform (version 3.0). If we consider 50 bp sequencing reads as an example, then we would expect that at least ≥5-10% of the sequencing capacity will be lost to sequence only the PCR primers (mean length of 20 bases). Sample pooling before target enrichment is possible using array/hybridization-based sequence capture methods and no loss in sequence capacity is expected, since the binding oligos/probes are kept fixed on the array and only the enriched genomic products are eluted from the array and sequenced [30]. The array-based sequence capture approach may however be limited with regard to selection of complete genomic regions due to repeat masking before designing certain capture probes.

As a final point, due to the knowledge of the exact start and stop position of each amplicon within the primer library, the necessary amount of sequencing required for a given sample can be precisely calculated, depending on the level of coverage that is required. For the 384 amplicon library used in this study, the sum total of amplicon bases was 172,805. The amount of sequencing required to achieve 100× average coverage, which results in >85% of the bases represented at a minimum coverage of 20×, is 17,280,500 bases. The total reads for the 6× pool (792L BCs 1–6) was 33,169,196 total reads and 7,221,473 reads on target, resulting in 361,073,648 bases per octet. This would have allowed up to 20 samples to be pooled per octet or 160 samples per flow cell and 320 samples per run on the SOLiD v3 system (Additional file 3: Table S7). Another practicable scenario to improve coverage for larger target regions could be using a lower degree of multiplexing (2-, 3-, 4-, or 5-plex scheme) on a quarter of a sequencing slide (quads; 4 well slide) instead of on an eighth of a slide (octant; 8 well slide). Following such a strategy would allow more sequencing room and subsequent higher coverage magnitude; by decreasing the inherent effect of slide’s physical separation that decreases the overall number of sequences obtained. In addition, recent rapid improvement of NGS technologies would in principle allow a higher level of sample multiplexing to bring per-sample cost down further.

## Conclusions

In this study, we have demonstrated the ability to combine PCR solution-based targeted sequencing and the high throughput of NGS to process samples that have been pooled and indexed at several different steps within the targeted sequencing workflow. For standard gDNA we found no significant difference in the sequence performance among the samples tested on the short-read ABI SOLiD platform: The sequence specificity (reads on target) was ~65%, the uniformity was ~85%, and the genotype concordance was 99%. Although we here did not test pooled WGA gDNA samples, the performance of our non-pooled WGA samples showed sufficient promise to merit more extensive investigations in the future. In summary, the ability to generate high quality and uniform sequence data across WGA and pooled gDNA samples using the described T-NGS approach may allow researchers to achieve the necessary statistical power within their studies to elucidate the underlying biology.

## Abbreviations

WGA: Whole Genome Amplification; NGS: Next Generation Sequencing; T-NGS: Targeted-NSG; GWAS: Genome-Wide Association Studies; gDNA: genomic Deoxyribonucleic Acid; emPCR: Emulsion Polymerase Chain Reaction; BC: Barcode; MAF: Minor allele frequency.

## Competing interests

James Brayer, Jason Warner, Jeff Olson and Darren Link are employees of RainDance Technologies.

## Authors’ contributions

AE, JB, AF designed research; JO, DL, JB performed sample enrichment using droplet-based PCR technology; AE, MBS, PR, AF conducted NGS sequencing; JW and MF performed data analysis and submission; AE, JW, JB interpreted the data; AE wrote the first draft of the manuscript; JB, JW, MF, MBS, PR, SS, SRJ., AF proof-read and edited the manuscript; AE, JB, SS, PR, AF coordinated the project. AE, JB revised the manuscript. All authors read and approved the final manuscript.

## Supplementary Material

Additional file 1 **Table S2.** An Overview of the RDT 384 Member Panel. Table includes individual tabs describing the amplicons, primers and gff. Click here for file

Additional file 2 **Figure S1.** RainDance Genomic DNA Template Droplet and Primer Droplet Merge. PCR droplets are generated on the RDT 1000 instrument (RainDance Technologies, Inc. USA). For a single sample, the gDNA template mix and the RainDance 384 Member Primer Library and four consumables are required, namely RDT 1000 Chip, RDT 1000 Template input/output vial, RDT 1000 Collect input/output vial, and a PCR Tube Strip, Axygen Scientific. The RDT 1000 instrument generated each PCR droplet by pairing a single gDNA template droplet with a single primer droplet. The paired droplets flow past an electrode embedded in the chip and are instantly merged creating the PCR droplet. All of the resulting PCR droplets were automatically dispensed as an emulsion into a single PCR tube and transferred to a standard thermal cycler for PCR amplification. **Figure S2.** A and B Comparison of DNA Fragment Distribution of RainDance 384 Member Primer Panel BioAnalyzer Trace with RainDance 384 Member Primer Library Predicted Amplicon Profile. Comparisons of the DNA fragment distribution of RainDance 384 Member Primer Panel Bioanalyzer Trace (2A) with the predicted profile (2B). As shown here, the amplicon profile obtained from the Agilent Bioanalyzer results (2A) nicely matches the predicted histogram distribution (2B). **Figure S3.** Removal of Primer-Dimer Peaks Using Agencourt AMPure Kit. (A) A typical electropherogram obtained showed a primer-dimer peak between 60 and 70 bp (~ 68 bp). B) The primer-dimer peak (and other unincorporated dNTPs, primers, salts and other contaminants) were removed after purification of the SOLiD Fragment Library (here Library 759L is shown as an example) using the standard procedure of Agencourt AMPure Kit (Beckman Coulter Genomics). **Figure S4.** Coverage Uniformity Across all the Barcoded and Pooled Samples Before (pB) and After (pA) Emulsion PCR. A comparable distribution of average depth of coverage (ADoC) across libraries pooled before and after emulsion PCR (emPCR) is shown here. Barcoded libraries pooled after emPCR (792 (pA)), showed more uniform ADoC. Samples assigned to barcode 4 (yellow point) showed the lowest ADoC. Click here for file

Additional file 3 **Table S1.** Overview of Sample Processing: RainDance Sequence Enrichment, SOLiD Sequencing Library Construction and Sample Indexing, Emulsion PCR and Sequencing. The samples were enriched on the RDT1000, followed by shearing and standard library construction according to the SOLiD 3.0 protocol (Applied Biosystems/ Life Technologies, USA). Six individual libraries were prepared using the enriched gDNA products and indexed with barcodes 1 to 6 (Library IDs 759L, 760L, 761L, 762L, 763L and 764L). Three additional libraries were prepared using the enriched WGA products and indexed with barcodes 9 to 11 (Library IDs 765L, 766L and 767L). To test the performance of each step, the non-amplified samples were pooled before and after the library preparation process. Pre-library preparation pools were created by combining an equimolar portion of the 6 individual gDNA. The samples were pooled together and processed as single samples (Library IDs 768_1L and 768_2L were duplicate libraries that contained six gDNA samples). Post-library preparation pools were created by combining equimolar portions of each individual sample post library preparation. A post library preparation pool was generated before emPCR (Emulsion ID 770em: gDNA samples 1, 2, 3, 4 and 6) and after emPCR (Emulsion ID 792em: gDNA samples 1, 2, 3, 4, 5 and 6). The resulting PCR products were sequenced on a SOLiD 3.0 system using 50 bp fragment libraries. **Table S3.** CLC Bio SNP Detection Parameters. The following parameters were used for SNP detection through the CLC bio Genomics Workbench (version 3.0): Maximum coverage 50000. Maximum gap and mismatch count 2. Minimum average quality 15. Minimum central quality 20. Minimum coverage 5. Minimum variant frequency (%) 10.0. Variant count threshold 50000. Window length 11. In the non-pooled samples, a SNP with a non-reference allele frequency of 10-90% was considered a heterozygote. A homozygous SNP in non-pooled samples was defined as having >90% non-reference allele frequency. **Table S4.** Coverage Metrics CLC bio Genomics Workbench (version 5.1). **Table S6.** Sequence Data Generated Using 454 FLX and Illumina of the same Target Regions (172kb/384 exons). **Table S7.** Sample Multiplexing Calculation for the RDT 384 Member Panel and SOLiD Sequencing Platform. Click here for file

Additional file 4 **Table S5.** An Overview of All SNPs and Genotypes Detected. Genotypes from non-barcoded pooled samples. Table includes both inferred and non-inferred genotypes. Click here for file
