# Supplementary material for: Accurate variant detection across non-amplified and whole genome amplified DNA using targeted next generation sequencing
Source: BMC Genomics. 2012 Sep 20;13:500. doi: 10.1186/1471-2164-13-500 (PMC3534403; doi:10.1186/1471-2164-13-500)
Supplement: Additional file 1 — Table S2. An Overview of the RDT 384 Member Panel. Table includes individual tabs describing the amplicons, primers and gff. [file 1471-2164-13-500-S1.pdf]

Supplementary Figures

Accurate Variant Detection across Non-amplified and Whole Genome Amplified DNA Using Targeted Next Generation Sequencing

Abdou ElSharawy[1], Jason Warner[2], Jeff Olson[2], Michael Forster[1], Markus B. Schilhabel[1], Darren Link[2], Stefan Rose-John[3], Stefan Schreiber[1,4], Philip Rosenstiel[1], James Brayer[2] and Andre Franke[1,*]

[1] Institute of Clinical Molecular Biology, Christian-Albrechts-University, Kiel, Germany

[2] RainDance Technologies, Inc. Lexington, Massachusetts, U.S.A.

[3] Institute of Biochemistry, Christian-Albrechts-University, Kiel, Germany

[4] First Medical Clinic, University Hospital, Schleswig-Holstein, Kiel, Germany

Email addresses

AE: a.sharawy@mucosa.de

JW: WARNERJ@raindancetech.com

JO: OLSONJ@raindancetech.com

MF: m.forster@ikmb.uni-kiel.de

MS: m.schilhabel@ikmb.uni-kiel.de

DL: dlink@raindancetechnologies.com

SR: rosejohn@biochem.uni-kiel.de

SS: s.schreiber@mucosa.de

PR: p.rosenstiel@mucosa.de

JP: brayerj@raindancetech.com

AF: a.franke@mucosa.de

Supplementary Figure 1

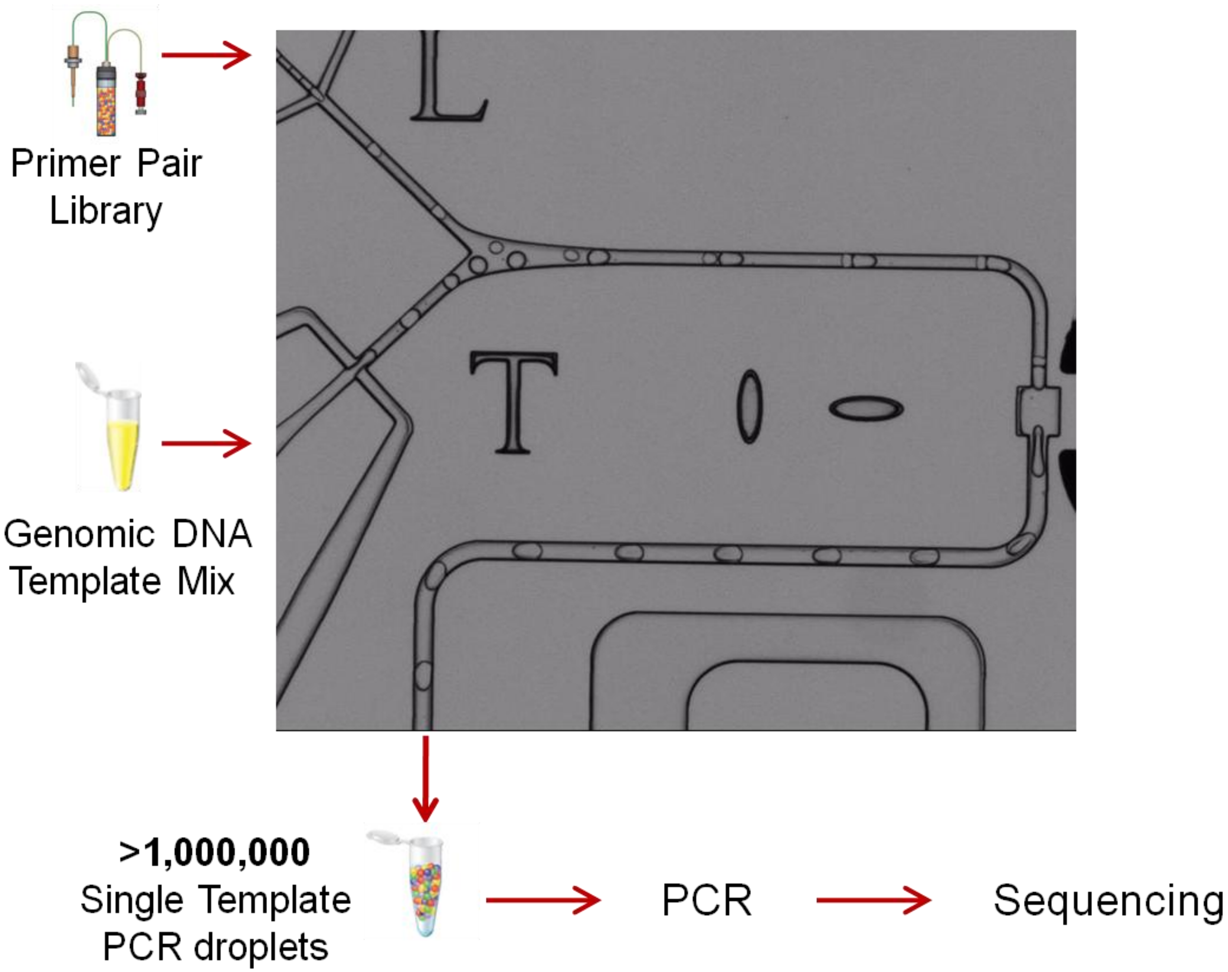

Supplementary Figure 2.A

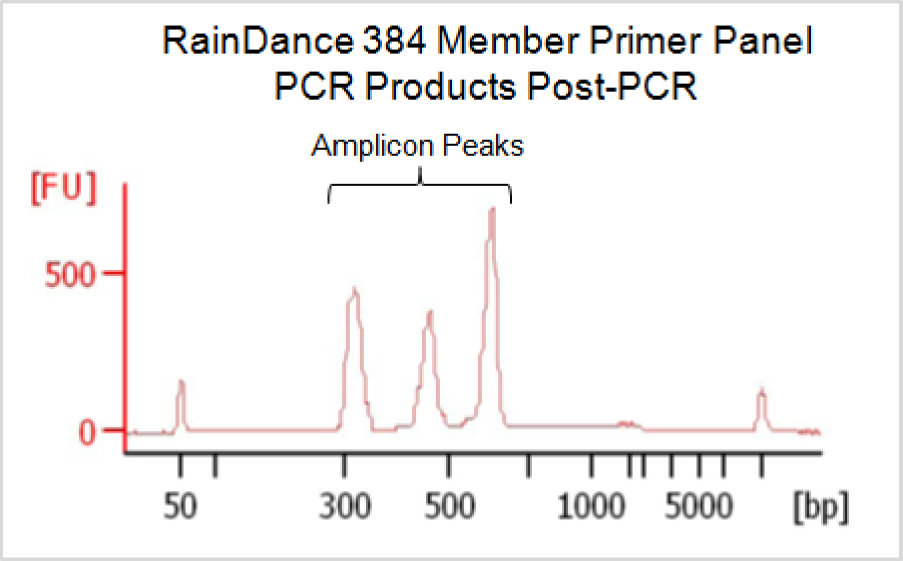

Supplementary Figure 2.B

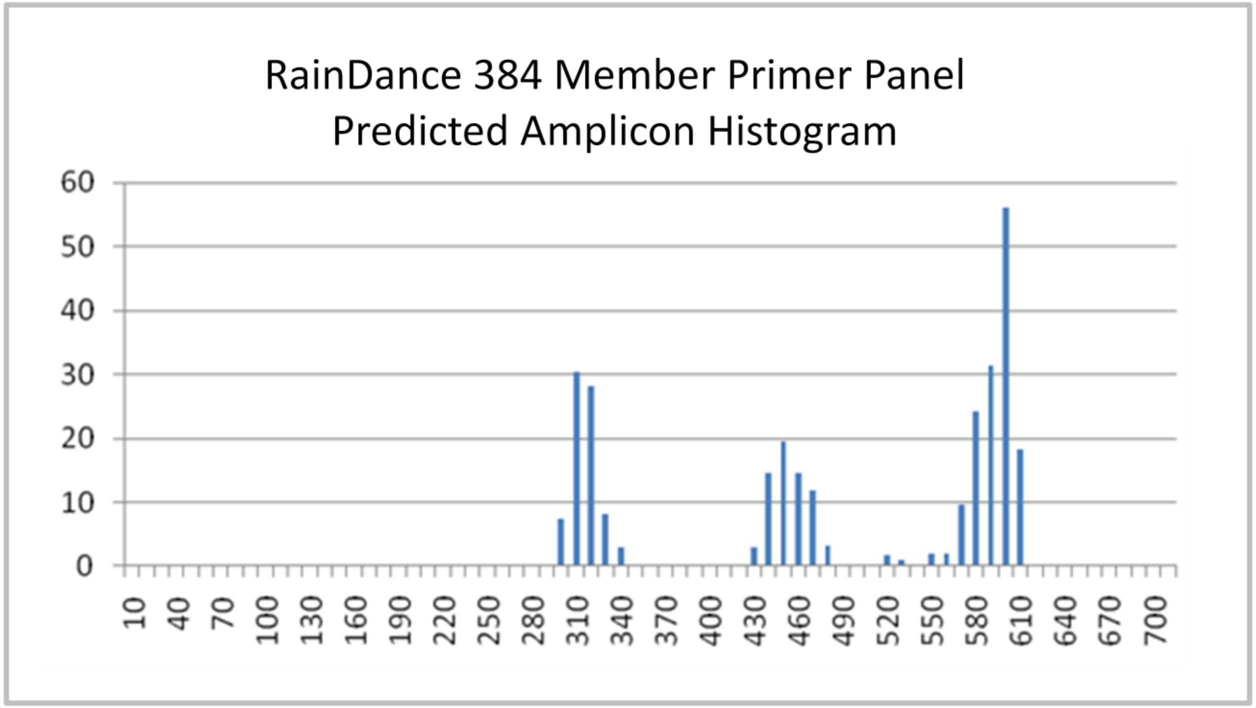

Supplementary Figure 3.A

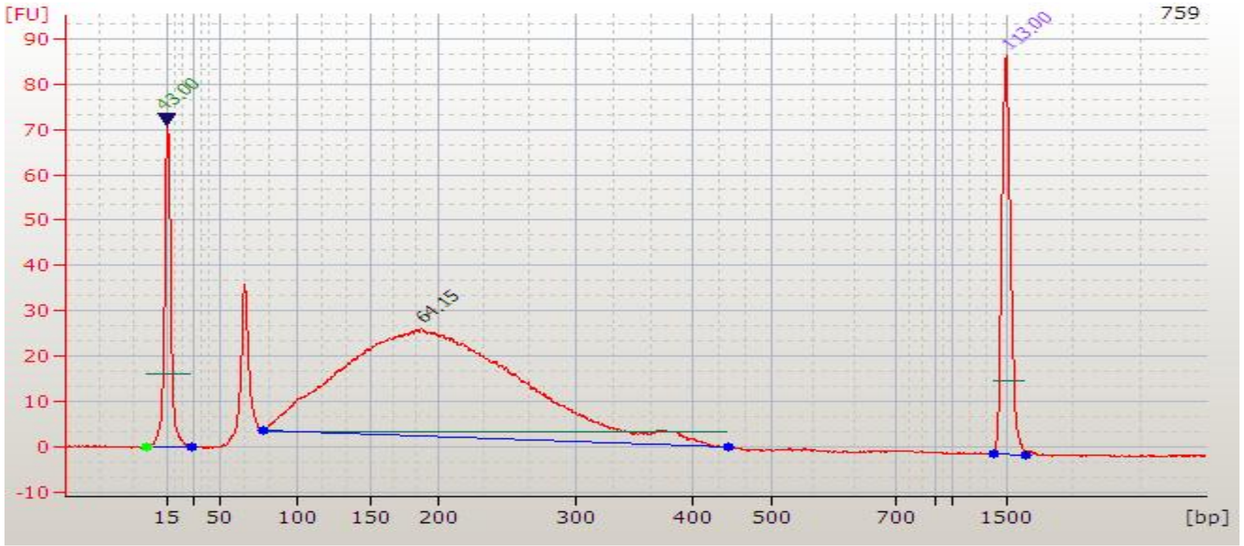

Supplementary Figure 3.B

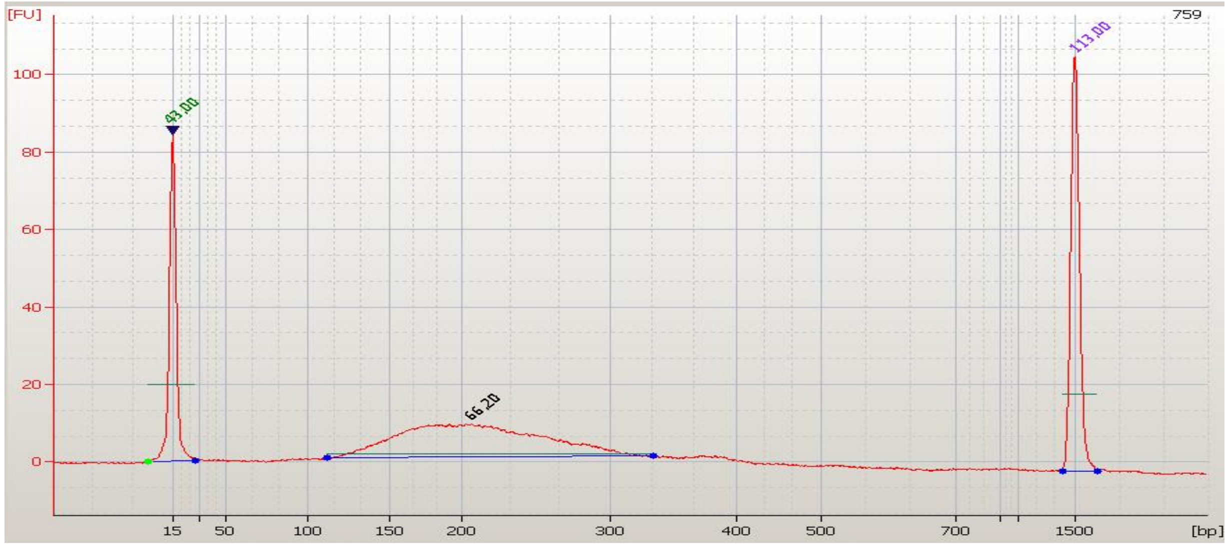

Supplementary Figure 4

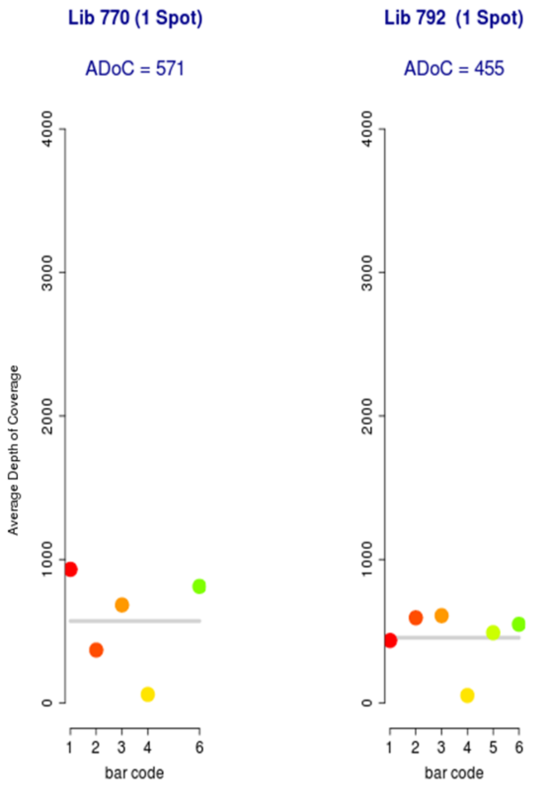

5 BC / spot gDNA Pooling *pre*-emPCR

6 BC / spot gDNA Pooling *post*-emPCR
