# Supplementary material for: Accurate variant detection across non-amplified and whole genome amplified DNA using targeted next generation sequencing
Source: BMC Genomics. 2012 Sep 20;13:500. doi: 10.1186/1471-2164-13-500 (PMC3534403; doi:10.1186/1471-2164-13-500)
Supplement: Additional file 2 — Figure S1. RainDance Genomic DNA Template Droplet and Primer Droplet Merge. PCR droplets are generated on the RDT 1000 instrument (RainDance Technologies, Inc. USA). For a single sample, the gDNA template mix and the RainDance 384 Member Primer Library and four consumables are required, namely RDT 1000 Chip, RDT 1000 Template input/output vial, RDT 1000 Collect input/output vial, and a PCR Tube Strip, Axygen Scientific. The RDT 1000 instrument generated each PCR droplet by pairing a single gDNA template droplet with a single primer droplet. The paired droplets flow past an electrode embedded in the chip and are instantly merged creating the PCR droplet. All of the resulting PCR droplets were automatically dispensed as an emulsion into a single PCR tube and transferred to a standard thermal cycler for PCR amplification. Figure S2. A and B Comparison of DNA Fragment Distribution of RainDance 384 Member Primer Panel BioAnalyzer Trace with RainDance 384 Member Primer Library Predicted Amplicon Profile. Comparisons of the DNA fragment distribution of RainDance 384 Member Primer Panel Bioanalyzer Trace (2A) with the predicted profile (2B). As shown here, the amplicon profile obtained from the Agilent Bioanalyzer results (2A) nicely matches the predicted histogram distribution (2B). Figure S3. Removal of Primer-Dimer Peaks Using Agencourt AMPure Kit. (A) A typical electropherogram obtained showed a primer-dimer peak between 60 and 70 bp (~ 68 bp). B) The primer-dimer peak (and other unincorporated dNTPs, primers, salts and other contaminants) were removed after purification of the SOLiD Fragment Library (here Library 759L is shown as an example) using the standard procedure of Agencourt AMPure Kit (Beckman Coulter Genomics). Figure S4. Coverage Uniformity Across all the Barcoded and Pooled Samples Before (pB) and After (pA) Emulsion PCR. A comparable distribution of average depth of coverage (ADoC) across libraries pooled before and after emulsion PCR (emPCR) is shown here [file 1471-2164-13-500-S2.pdf]

# Supplementary Tables

## Accurate Variant Detection across Non-amplified and Whole Genome Amplified DNA Using Targeted Next Generation Sequencing

Abdou ElSharawy[1], Jason Warner[2], Jeff Olson[2], Michael Forster[1], Markus B. Schilhabel[1], Darren Link[2], Stefan Rose-John[3], Stefan Schreiber[1,4], Philip Rosenstiel[1], James Brayer[2] and Andre Franke[1,*]

[1] Institute of Clinical Molecular Biology, Christian-Albrechts-University, Kiel, Germany

[2] RainDance Technologies, Inc. Lexington, Massachusetts, U.S.A.

[3] Institute of Biochemistry, Christian-Albrechts-University, Kiel, Germany

[4] First Medical Clinic, University Hospital, Schleswig-Holstein, Kiel, Germany

Email addresses

AE: a.sharawy@mucosa.de

JW: WARNERJ@raindancetech.com

JO: OLSONJ@raindancetech.com

MF: m.forster@ikmb.uni-kiel.de

MS: m.schilhabel@ikmb.uni-kiel.de

DL: dlink@raindancetechnologies.com

SR: rosejohn@biochem.uni-kiel.de

SS: s.schreiber@mucosa.de

PR: p.rosenstiel@mucosa.de

JP: brayerj@raindancetech.com

AF: a.franke@mucosa.de

**Supplementary Table 1: Overview of Sample Processing: RainDance Sequence Enrichment, SOLiD Sequencing Library Construction and Sample Indexing, Emulsion PCR and Sequencing**

| Sample ID | HapMap Sample_ID | Family ID | Library ID | Bar Code | Emulsion ID | Sequencing ID |
|---|---|---|---|---|---|---|
| 1 | NA12003 (gDNA) | 1420 | 759L | 1 | 759em | 759seq |
| 2 | NA12004 (gDNA) | 1420 | 760L | 2 | 760em | 760seq |
| 3 | NA10838 (gDNA) | 1420 | 761L | 3 | 761em | 761seq |
| 4 | NA11829 (gDNA) | 1350 | 762L | 4 | 762em | 762seq |
| 5 | NA11830 (gDNA) | 1350 | 763L | 5 | 763em | 763seq |
| 6 | NA10856 (gDNA) | 1350 | 764L | 6 | 764em | 764seq |
| 1-6_1 | ***6 DNAs pooled before library preparation*** | 1350 and 1420 | 768_1L | - | 768_1Lem | 768_1Lseq |
| 1-6_2 | ***6 DNAs pooled before library preparation**** | 1350 and 1420 | 768_2L | - | 768_2Lem | 768_2Lseq |
| 7 | ***Indexed Sample: pooled before emPCR (pB)*** | 1350 and 1420 | 770L | 1-4 & 6 | 770em | 770seq |
| 8 | ***Indexed samples: pooled after emPCR (pA)*** | 1350 and 1420 | 792L | 1-6 | 792em | 792seq |
| 9 | NA12003 (WGA gDNA) | 1420 | 765L | 9 | 765em | 765seq |
| 10 | NA12004 (WGA gDNA) | 1420 | 766L | 10 | 766em | 766seq |
| 11 | NA10838 (WGA gDNA) | 1420 | 767L | 11 | 767em | 767seq |

* Technical replicate of library 786_1.
- Boldfaced italic words indicate pooled samples/libraries.
- gDNA: genomic DNA; WGA: whole-genome amplification; emPCR: emulsion PCR.

**Supplementary Table 2: RainDance Technologies 384 Member Primer Panel - gff**

**(A separate Excel sheet)**

**Supplementary Table 3: CLC Bio SNP Detection Parameters**

| | |
|---|---|
| | |
| Add conflict annotations | No |
| Alignment mode | local |
| Amplicon annotation | |
| Annotate consensus sequence | Yes |
| Annotate reference sequence | Yes |
| Colorspace alignment | Yes |
| Colorspace Alignment | Yes |
| Colorspace error cost | 3 |
| Colorspace Error Cost | 3 |
| Conflict resolution Vote | |
| Create Report | Yes |
| Create SequenceList | Yes |
| Create table | Yes |
| Deletion Cost | 3 |
| Genetic code translation | Standard |
| Guidance only | No |
| Insertion cost | 3 |
| Insertion Cost | 3 |
| Length | 0.5 |
| Mask reference sequence | |
| Match mode random | |
| Max alignments | 1 |
| Max distance | 250 |
| Maximum coverage | 50000 |
| Maximum expected variations (ploidy) | 2 |
| Maximum gap and mismatch count | 2 |
| Min distance | 180 |

| | |
|---|---|
| Minimum average quality | 15 |
| Minimum central quality | 20 |
| Minimum coverage | 5 |
| Minimum paired-end coverage | 0 |
| Minimum variant frequency (%) | 10.0 |
| Mismatch cost | 2 |
| Mismatch Cost | 2 |
| Read settings | |
| Reference sequences | chr21, chr22, chrY, chr20, chr19, chr18, chr17, chr16, chr15, chr14, chr13, chr12, chr11, chr10, chr9, chr8, chrX, chr7, chr6, chr5, chr4, chr3, chr2, chr1 |
| Report type | 1x, 5x, 10x, 20x, 40x, 80x, 100x |
| Score limit | 8 |
| Sequence masking | No |
| Similarity | 0.8 |
| Ungapped alignment | Yes |
| Use annotated amplicons | No |
| Variant count threshold | 50 |
| Window length | 11 |

## Supplementary Table 4: Coverage Metrics CLC bio Genomics Workbench (version 5.1)

| HapMap Sample ID | Sample Type | Library ID | Reads | Mapped | | On-Target % | | ADoC | C1 | C10 | C20 | C30 | C50 | C100 | Coverage 0.2X Mean |
|---|---|---|---|---|---|---|---|---|---|---|---|---|---|---|---|
| NA12003 | gDNA | 759L | 36,453,208 | 19,290,598 | 52.92% | 8,577,722 | 44.47% | 2309.47 | 99.43% | 98.47% | 98.15% | 97.92% | 97.39% | 96.24% | 87.49% |
| | WGA | 765L | 36,986,943 | 20,508,825 | 55.45% | 9,361,663 | 45.65% | 2541.34 | 99.41% | 98.56% | 98.15% | 97.83% | 97.34% | 96.54% | 86.97% |
| | gDNA-pB | 770L_BC1 | 10,031,809 | 5,627,776 | 56.10% | 2,489,710 | 44.24% | 671.32 | 98.82% | 97.75% | 96.79% | 96.16% | 94.66% | 90.56% | 87.63% |
| | gDNA-pA | 792L_BC1 | 5,221,822 | 2,678,148 | 51.29% | 1,142,288 | 42.65% | 302.78 | 98.37% | 96.43% | 94.70% | 92.87% | 89.08% | 77.89% | 87.03% |
| NA12004 | gDNA | 760L | 39,005,646 | 19,804,791 | 50.77% | 9,852,432 | 49.75% | 2606.79 | 99.25% | 98.37% | 98.03% | 97.76% | 97.37% | 96.35% | 87.66% |
| | WGA | 766L | 43,079,257 | 16,264,344 | 37.75% | 6,035,720 | 37.11% | 1515.41 | 99.22% | 98.01% | 97.45% | 97.17% | 96.55% | 94.84% | 86.28% |
| | gDNA-pB | 770L_BC2 | 3,272,424 | 1,883,818 | 57.57% | 987,460 | 52.42% | 266.77 | 98.17% | 96.45% | 94.75% | 92.74% | 88.77% | 76.85% | 88.16% |
| | gDNA-pA | 792L_BC2 | 6,023,113 | 3,088,315 | 51.27% | 1,546,582 | 50.08% | 408.18 | 98.40% | 96.94% | 95.64% | 94.33% | 91.75% | 84.79% | 87.41% |
| NA10838 | gDNA | 761L | 35,573,703 | 14,695,823 | 41.31% | 5,705,050 | 38.82% | 1481.41 | 99.24% | 98.20% | 97.73% | 97.27% | 96.55% | 94.89% | 87.12% |
| | WGA | 767L | 43,140,968 | 21,584,861 | 50.03% | 8,194,931 | 37.97% | 2189.59 | 99.42% | 98.54% | 98.14% | 97.80% | 97.16% | 96.04% | 87.69% |
| | gDNA-pB | 770L_BC3 | 7,114,637 | 3,869,662 | 54.39% | 1,809,019 | 46.75% | 489.74 | 98.70% | 97.19% | 95.95% | 94.97% | 92.83% | 86.75% | 87.12% |
| | gDNA-pA | 792L_BC3 | 6,587,601 | 3,489,971 | 52.98% | 1,600,827 | 45.87% | 430.28 | 98.54% | 97.00% | 95.71% | 94.49% | 91.72% | 85.08% | 87.00% |
| NA11829 | gDNA | 762L | 39,864,620 | 18,798,516 | 47.16% | 7,830,282 | 41.65% | 2099.65 | 99.47% | 98.47% | 98.09% | 97.70% | 97.15% | 96.00% | 87.03% |
| | gDNA-pB | 770L_BC4* | 638,985 | 354,599 | 55.49% | 161,742 | 45.61% | 43.75 | 95.99% | 83.63% | 66.47% | 49.70% | 28.12% | 9.87% | 86.54% |

| | | | | | | | | | | | | | | |
|---|---|---|---|---|---|---|---|---|---|---|---|---|---|---|
| | **gDNA-pA** | **792L_BC4*** | 677,767 | 335,666 | 49.53% | 139,652 | 41.60% | 36.98 | 95.38% | 80.11% | 59.39% | 41.17% | 22.45% | 6.88% | 85.65% |
| **NA11830** | **gDNA** | **763L** | 38,654,830 | 20,144,033 | 52.11% | 9,425,152 | 46.79% | 2557.17 | 99.53% | 98.61% | 98.21% | 97.92% | 97.42% | 96.45% | 87.07% |
| | **gDNA-pB** | **770L_BC5** | Insufficient amount of material to run sample | | | | | | | | | | | | |
| | **gDNA-pA** | **792L_BC5** | 5,403,560 | 2,832,411 | 52.42% | 1,296,850 | 45.79% | 346.19 | 98.38% | 96.87% | 95.24% | 93.64% | 90.28% | 81.46% | 86.91% |
| **NA10856** | **gDNA** | **764L** | 43,185,707 | 22,650,137 | 52.45% | 9,489,382 | 41.90% | 2574.94 | 99.53% | 98.68% | 98.24% | 98.01% | 97.58% | 96.55% | 86.90% |
| | **gDNA-pB** | **770L_BC6** | 9,255,333 | 5,136,664 | 55.50% | 2,156,020 | 41.97% | 584.63 | 98.90% | 97.62% | 96.70% | 95.74% | 93.93% | 89.00% | 87.30% |
| | **gDNA-pA** | **792L_BC6** | 6,435,690 | 3,407,875 | 52.95% | 1,423,554 | 41.77% | 381.03 | 98.64% | 96.96% | 95.54% | 93.84% | 90.87% | 82.48% | 86.57% |
| **Pooled Samples** | **gDNA** | **768L_1** | 41,913,860 | 21,748,402 | 51.89% | 9,833,007 | 45.21% | 2627.27 | 99.55% | 98.63% | 98.22% | 98.05% | 97.67% | 96.85% | 87.01% |
| | **gDNA** | **768L_2** | 41,958,628 | 22,079,022 | 52.62% | 10,073,995 | 45.63% | 2700.44 | 99.47% | 98.66% | 98.25% | 98.08% | 97.69% | 96.90% | 87.25% |

**Supplementary Table 5: An overview of all SNPs and genotypes detected**

**(A separate Excel sheet)**

**Supplementary Table 6: Sequence Data Generated using 454 FLX and Illumina of the Same Target Regions (172kb/384 exons)**

| **Targeted Sequencing Results** | **454 FLX** | **Illumina** |
|---|---|---|
| Total Reads | 219,876 | 5,954,822 |
| Reads Mapping to Target Regions | 184,641 | 2,337,390 |
| Average Mapped Reads per Amplicon | 482 | 5,343 |
| Percent of Target Amplicons Covered | 99.7% | 99.7% |
| Target Uniformity (<10-fold) | 96% | 98% |

## Supplementary Table 7: Sample Multiplexing Calculation

| Library ID | Bar-code | Reads | Mapped to Genome | Mapped on Target |
|---|---|---|---|---|
| 792L | BC1 | 5,221,822 | 31.60% | 64.20% |
| | BC2 | 6,023,113 | 32.50% | 72.90% |
| | BC3 | 6,587,601 | 34.00% | 67.60% |
| | BC4 | 677,767 | 28.50% | 66.40% |
| | BC5 | 5,403,560 | 32.80% | 68.10% |
| | BC6 | 9,255,333 | 36.30% | 61.30% |
| | Total Reads (BC1-BC6) | **33,169,196** | | |
| | Average (BC1-BC6) | | **32.62%** | **66.75%** |
| | | | | |
| Total Reads per Octet | A | 33,169,196 | | |
| Average % of Reads Mapped to Genome | B | 32.62% | | |
| Mapped Reads | C | 10,818,686 | = A*B | |
| % of read on Target | D | 66.75% | | |
| Reads on Target | E | 7,221,473 | = C*D | |
| Read length (bases) | F | 50 | | |
| Bases per Octet | G | 361,073,648 | = E*F | |
| Bases per Sample* | H | 17,280,500 | | |
| Samples per Octet | I | 20 | = G/H | |
| Sample per Flow Cell | J | 160 | = I*8 | |
| Samples per Run | K | 320 | = I*16 | |

*Bases per Sample = # of amplicon bases * 100
